# The electrophysiological connectome is maintained in healthy elders: a power envelope correlation MEG study

**DOI:** 10.1038/s41598-017-13829-8

**Published:** 2017-10-25

**Authors:** N. Coquelet, A. Mary, P. Peigneux, S. Goldman, V. Wens, X. De Tiège

**Affiliations:** 1 Laboratoire de Cartographie fonctionnelle du Cerveau (LCFC), UNI – ULB Neuroscience Institute, Université libre de Bruxelles (ULB), Brussels, Belgium; 2 Neuropsychology and Functional Imaging Research Group (UR2NF), Centre for Research in Cognition and Neurosciences (CRCN), UNI – ULB Neuroscience Institute, Université libre de Bruxelles (ULB), Brussels, Belgium; 3 Normandie Univ, UNICAEN, PSL Research University, EPHE, INSERM, U1077, CHU de Caen, Neuropsychologie et Imagerie de la Mémoire Humaine, Caen, France; 4 Department of functional Neuroimaging, CUB-Hôpital Erasme, Université libre de Bruxelles, Brussels, Belgium

## Abstract

Functional magnetic resonance imaging (fMRI) studies report age-related changes in resting-state functional connectivity (rsFC), suggesting altered or reorganized connectivity patterns with age. However, age-related changes in neurovascular coupling might also partially account for altered connectivity patterns. Here, we used resting-state magnetoencephalography (MEG) and a connectome approach in carefully selected healthy young adults and elders. The MEG connectome was estimated as rsFC matrices involving forty nodes from six major  resting-state networks. Source-level rsFC maps were computed in relevant frequency bands using leakage-corrected envelope correlations. Group differences were statistically assessed using non-parametric permutation tests. Our results failed to evidence significant age-related differences after correction for multiple comparisons in the α and the β bands both for static and dynamic rsFC, suggesting that the electrophysiological connectome is maintained in healthy ageing. Further studies should compare the evolution of the human brain connectome as estimated using fMRI and MEG in same healthy young and elder adults, as well as in ageing conditions associated with cognitive decline. At present, our results are in agreement with the brain maintenance theory for successful aging as they suggest that preserved intrinsic functional brain integration contributes to preserved cognitive functioning in healthy elders.

## Introduction

Substantial changes in sensory perception^1–3^, motor^4^ and cognitive abilities (for a review, see, e.g.^5^) usually occur with age. Nevertheless, there is a marked inter-individual variability in the age-related decline of brain functions, and particularly regarding cognitive functioning (for reviews, see, e.g.^6,7^). Indeed, in a population of subjects with no characterized neurodegenerative disorders, some individuals may show early (i.e., in their 50 s) reliable decline, while others may show late (i.e., in their 70 s or even 80 s) preserved functioning^6,7^. The neural correlates subtending age-related behavioural and cognitive decline have been a major topic of neuroimaging research these last decades. Conversely, there has been  increased interest in the last years in  the brain mechanisms subtending “healthy” or “successful” aging^6^.

Structurally, physiological aging is usually considered to be associated with changes in brain volume and cerebrospinal fluid spaces^8–12^ that are due to progressive regional grey matter and more widespread white matter loss^13^. White matter loss induces a disruption of fiber tracks connecting specific large-scale neural network nodes involved in high-level brain functions^14–16^. Interestingly, some structural magnetic resonance imaging (MRI) and diffusion tensor imaging (DTI) studies have suggested that the less structural brain changes are observed, the better is the cognitive functioning at old age (see, e.g^17–20^). These findings provide empirical support to the “*brain maintenance*” theory, which postulates that individual differences in the manifestation of age-related brain changes and pathology account for the variability in age-related cognitive decline^6^.

The first attempts to characterize the age-related modifications in the functional organization of the human brain during healthy aging have been performed using task-based functional MRI (fMRI) (for a review, see, e.g.^21^). A meta-analysis of task-related fMRI datasets has disclosed age-related hypo-activation of the visual areas and hyper-activations mainly in two cognitive networks, i.e., the fronto-parietal control (FPN) and the default mode (DMN) networks^21^. The latter finding was interpreted as an over-recruitment mechanism playing a crucial role in generating successful cognitive compensation in older adults^21^. Cerebral over-recruitment in aging provided some support to the “*cognitive reserve*” or the “*compensation*” hypotheses, which posit that some individuals will cope better with age-related pathology than others because they do react or compensate better for their progressive brain changes (for reviews, see, e.g.^22,23^).

A major disadvantage of task-based investigations is the possible performance biases between young adults and elder subjects in cross-sectional studies, or between elders with different behavioral and cognitive functioning. This indeed complicates the interpretation of age-related fMRI changes. Thus, with the advent of resting-state neuroimaging, there has been a growing interest in resting-state investigations to provide an accurate picture of the age-related changes in the functional human brain architecture, free of task-performance bias. Indeed, fMRI studies have shown that task-based brain networks configuration is actually shaped primarily by an intrinsic network architecture that is also present during the so-called “resting state” (i.e., in the absence of any explicit input or output)^24,25^. Such intrinsic network architecture emerges from the spontaneous low-frequency fluctuations of the blood-oxygen-level-dependent (BOLD) signal captured by fMRI (for a review, see, e.g.^26^).

In their seminal study, Andrews-Hanna *et al*.^27^ studied using resting-state fMRI the effect of aging on resting-state functional connectivity (rsFC). They highlighted a loss of rsFC between nodes of two important large-scale brain networks (i.e., the DMN and the dorsal attentional network (DAN)) with aging, and especially between the anterior and the posterior nodes of the DMN (i.e., between the medial prefrontal (mPFC) and the posterior cingulate (PCC) cortices). Importantly, the within-DMN rsFC positively correlated with the cognitive performance (i.e., executive, memory and processing speed) in older participants. However, the DAN and the DMN are not the sole networks impacted by aging, and age-related effects on other networks have also been (inconsistently) uncovered in subsequent fMRI studies. Resting-state fMRI investigations also found decreased rsFC in the FPN and cingulo-opercular networks, as well as increased in rsFC in somatosensory and subcortical networks (see, e.g.^28,29^). Besides within-network investigations, the effects of aging on cross-networks interactions were also investigated using fMRI and, e.g., disclosed increased interactions between the somatomotor and the visual networks with age^29^. Taken together, these studies and others (for reviews, see, e.g.^23,30^) demonstrated that normal aging impacts the intrinsic functional architecture of the human brain with either decreased or increased functional connectivity between nodes of the different networks. Some of these functional connectivity changes correlated with some cognitive measures, suggesting a direct link with the cognitive decline that can be observed with aging. These findings also partly bring support to the “*cognitive reserve*” or the “*compensation*” hypotheses.

When it turns to the study of the age-related changes in functional brain architecture using fMRI, two important issues should be considered. First, aging is frequently associated with an increased prevalence of sleep disorders (e.g., insomnia), psychiatric conditions (e.g., anxiety, depression), or with the use of psychotropic drugs (e.g., sedatives) that can potentially influence rsFC estimates and bias the results (see, e.g.^31–33^). Second, fMRI actually provides indirect information about neuronal activity, by measuring the local variations of brain perfusion associated with changes in neuronal activity via the BOLD signal. The neurovascular coupling is considered to be altered with aging and some brain disorders (for a review, see, e.g.^34^). In this respect, the impact of age-related neurovascular coupling changes on rsFC measures as indexed by fMRI remains unsettled (for a review, see, e.g.^35^). Contrary to fMRI, magnetoencephalography (MEG) provides a direct measure of neuronal activity^36^ and allows bypassing this neurovascular coupling issue. Interestingly, networks similar to those observed with fMRI at rest, also called resting-state networks (RSNs), were uncovered using MEG from large-scale correlation patterns in the slow fluctuations of band-limited sources envelope (i.e., power envelope correlation), particularly in the alpha and the beta frequency bands^37–39^. Indeed, some RSNs emerge preferentially either in the alpha band (e.g., the visual network and the DMN) or in the beta band (e.g., the somatomotor or the auditory networks)^37,39^. These findings paved the way for the investigation of the electrophysiological bases of fMRI RSNs^37,39^. Furthermore, the excellent temporal resolution of the MEG, of the order of 1 ms, allows better investigation of the spatial, temporal and spectral dynamics of the human brain rsFC than fMRI. Previous MEG studies have indeed demonstrated that RSNs actually alternate between short periods (i.e., from hundred milliseconds to several seconds) of high correlation among nodes within or between RSNs, and periods during which only a subset of network nodes interact^40–42^. This dynamic functional integration within and between RSNs within specific frequency bands therefore appears as a key element of the intrinsic functional organization of the human brain.

In this study, we aimed at investigating further the age-related changes in functional brain integration using MEG rsFC in carefully selected elder participants, to ensure that they were free of any confounding factors that could affect MEG rsFC estimates. Also, in order to provide a more complete description of brain rsFC changes with aging, we investigated both static and dynamic MEG rsFC using the power envelope correlation approach. We explored within- and cross-networks interactions on the basis of a connectome matrix adapted from^41^. This latter approach relied on forty cortical nodes distributed across six major RSNs, i.e., the DMN, the DAN, the ventral attentional network (VAN), the visual network (VISN), the somatomotor network (SMN) and the language network (LAN). Most of these RSNs were shown to display significant changes in within- and between-RSNs rsFC in previous fMRI studies (see above). The reasons guiding the use of those methods were (i) that focusing on power envelope correlation as an index of brain rsFC would allow (by contrast with phase coupling approaches) to compare the MEG findings with the available fMRI literature, (ii) that dynamic rsFC would potentially detect subtle changes in the functional brain integration associated with aging, and (iii) that the connectome approach is a computationally efficient method limiting data analysis to a subset of relevant cortical nodes and, therefore, making interactions between nodes clearly readable. As we focused on power envelope correlation for rsFC computation, we also assessed the age-related changes in static and dynamic MEG power across the different RSNs nodes because previous studies have disclosed age-related changes in band-specific regional power (see, e.g.^43–46^). Based on the available literature, we expected to find substantial age-related changes in band-specific power and rsFC with both increases and decreases in the power of some frequency bands, and in within- and between-RSNs interactions. We also expected that dynamic band-specific power and rsFC would provide an additional and potentially more sensitive view than static approaches on the age-related changes in functional brain integration.

## Results

Neuromagnetic activity was recorded at rest (eyes opened) during 5 minutes using whole-scalp-covering MEG device in twenty-five young (12 females and 13 males; age: 23.6 ± 2.9 years (mean ± standard deviation)) and twenty-five elderly (15 females and 10 males; 68.8 ± 2.4 years) right-handed healthy adult subjects. All participants had no prior history of neurological or psychiatric disorder, and did not report any subjective sleep or cognitive (e.g., memory impairment) problem. More specifically, all elder subjects had active personal and social life, and were free of psychotropic drug intake as well as of known sleep problem, depression, anxiety and signs of pathological cognitive decline (see Table 1).

**Table 1:** Demographic data and scores for the elder population.

	Study n°1			Study n°2		
Number of participants (female)	15 (9)			10 (6)		
Age (years)	68.8 ± 1.6			68.8 ± 3.3		
**Tests**	**Scores**	**Range**	**Inclusion**	**Scores**	**Range**	**Inclusion**
Beck Depression Inventory	2.1 ± 2.3	0–7	≤7	n. a.	n. a.	n. a.
Geriatric Depression Scale	n. a.	n. a.	n. a.	0.6 ± 0.9	0–3	<5
STAI: A-State	25.5 ± 5.4	20–36	≤45	23.4 ± 6.2	20–39	≤45
STAI: A-Trait	31.9 ± 6.1	24–40	≤45	28.6 ± 7.5	22–47	≤45
Mattis Dementia Scale	141.5 ± 1.7	137–144	>123	n. a.	n. a.	n. a.
Montreal Cognitive Assessment	n. a.	n. a.	n. a.	29.4 ± 0.8	28–30	>26

Age and scores are represented as mean ± standard deviation.

**Figure 1:**
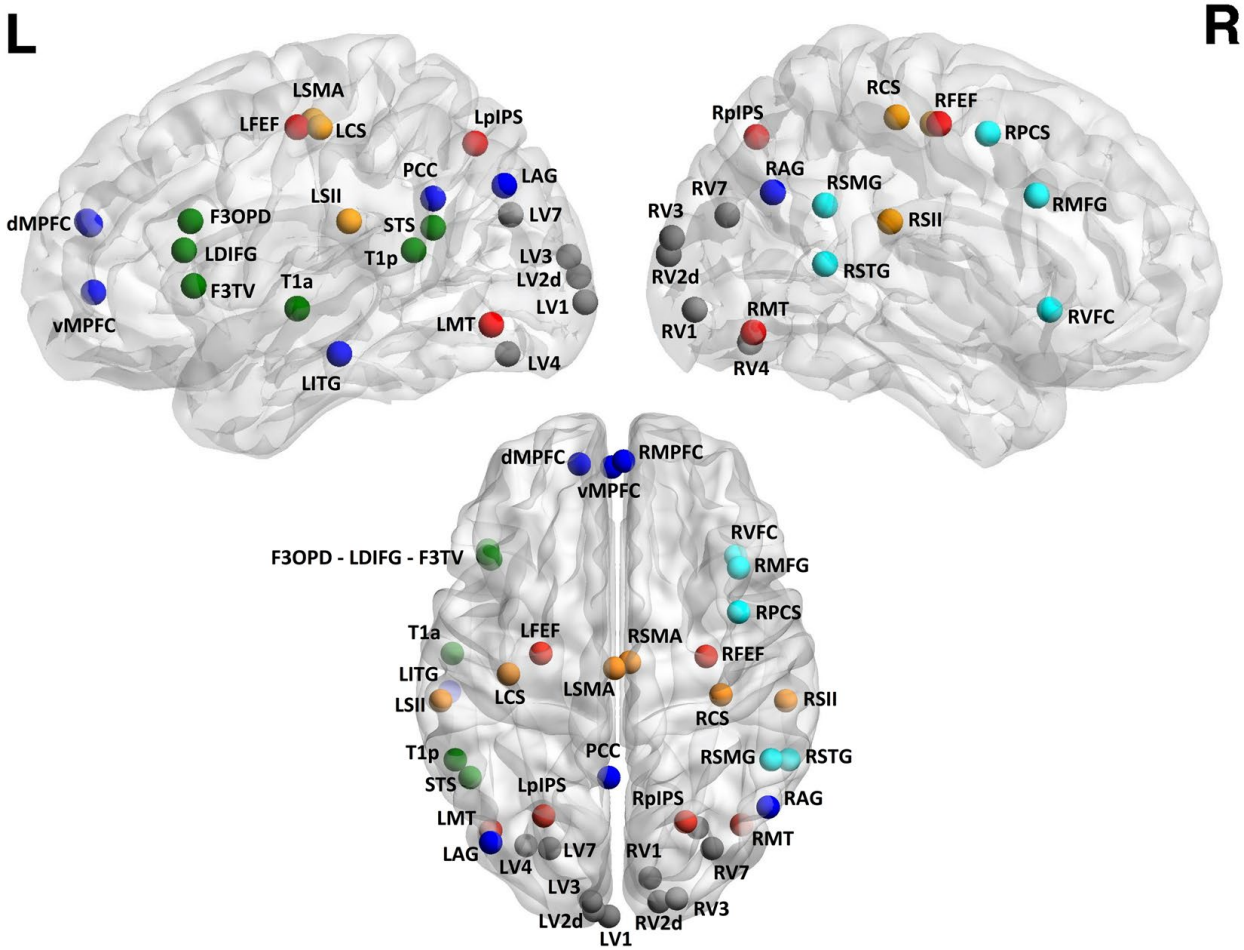
Locations and labels onto the glass MNI brain of the fourty cortical nodes considered in this study. The top parts correspond to the left (Left) and the right (Right) external faces of the hemispheres. The bottom part provides a view of the glass MNI brain from the top. Color code: red is associated with the DAN nodes, light blue with VAN nodes, dark blue with DMN nodes, gray with VISN nodes, orange with SMN nodes and green with LAN nodes. Coordinates and labels abbreviations  may be found in^41^.

The analysis of MEG rsFC was derived from source-reconstructed band-limited α (8–13 Hz) and β (13–25 Hz) power envelope correlations with geometric correction for spatial leakage effects^47^ at 40 cortical nodes belonging to 6 RSNs detectable by MEG^41^. Figure 1 displays the location of the 40 cortical nodes on the MNI brain. Both static and dynamic rsFC were considered, the former being computed over the whole 5 min MEG recording and the latter, within short (length: 10 s, steps: 2 s) time windows sliding along the recording^40,41^. To assess how the temporal variability of rsFC time series was affected by physiological aging, we considered the standard deviation (SD) and the coefficient of stability (CS, i.e., mean-over-SD) of the dynamic data, both computed in each individual across windows. We also derived α- and β-band static and dynamic power (with proper correction for depth bias) at these nodes to control for possible power-induced effects on rsFC. Furthermore, we computed static power in δ (1–3 Hz) and θ (4–7 Hz) frequency bands as previous studies have demonstrated changes in the power of those frequency bands with aging^43–46^. Group-level differences in rsFC or power entries between young and elder participants were evaluated statistically using non-parametric permutation testing. The large number of multiple comparisons involved in the analyses was taken into account by controlling the false discovery rate (FDR).

No age-related difference in static and dynamic rsFC within the α and the β bands survived the FDR correction. On this account, we cannot report differences in band-specific rsFC data between young and elder subjects.

**Figure 2:**
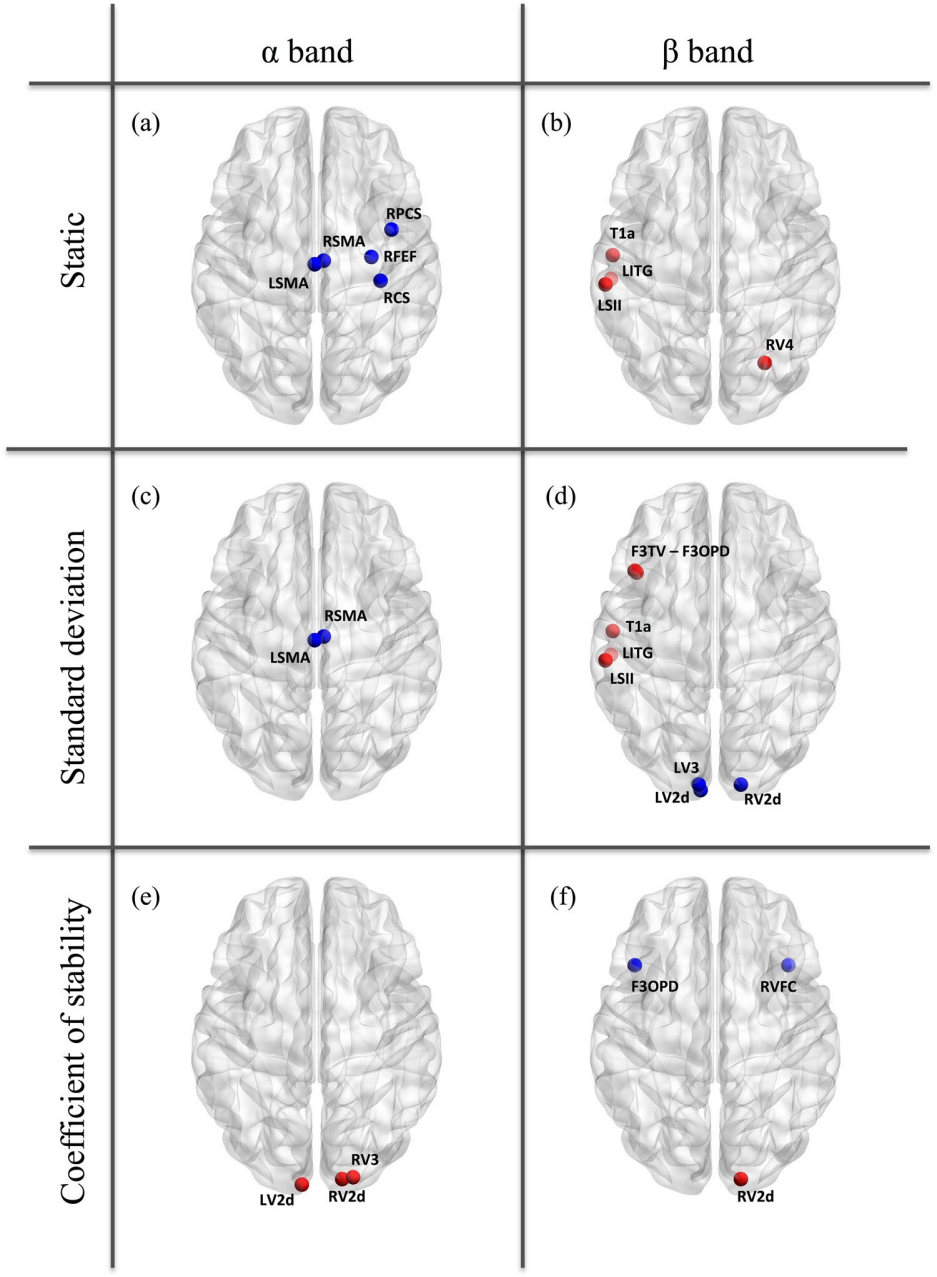
Significant age-related power increases (red) and decreases (blue) from young to elder subjects in the α band (left column) and the β band (right column), for the static (top), dynamic SD (middle) and dynamic CS (bottom) analyses. The nodes are displayed onto the glass MNI brain.

By contrast, we identified significant age-related power modulations both in the α and the β bands (Fig. 2). For the static evaluation, significant power decreases were found from young adults to elders for one node of the DAN, one node of the VAN, and two nodes of the SMN in the α band (Fig. 2a). Significant power increases were also disclosed for one node of the DMN, the LAN, the SMN, and the VISN in the β band (Fig. 2b). For the dynamic SD (Fig. 2c,d), significant age-related power decreases were observed for two SMN nodes in the α band and for three VISN nodes in the β band, while significant power increases were found in the β band only for 3 nodes in the LAN, one node in the SMN and one node in the DMN. For the CS, power increases in three nodes of the VISN were highlighted in the α band (Fig. 2e). The β-band CS displayed one power increase in the VISN and two power decreases in one node of LAN and one node of the VAN (Fig. 2f). The p-values associated with each age-related change are listed in the Supplementary Table S1.

**Figure 3:**
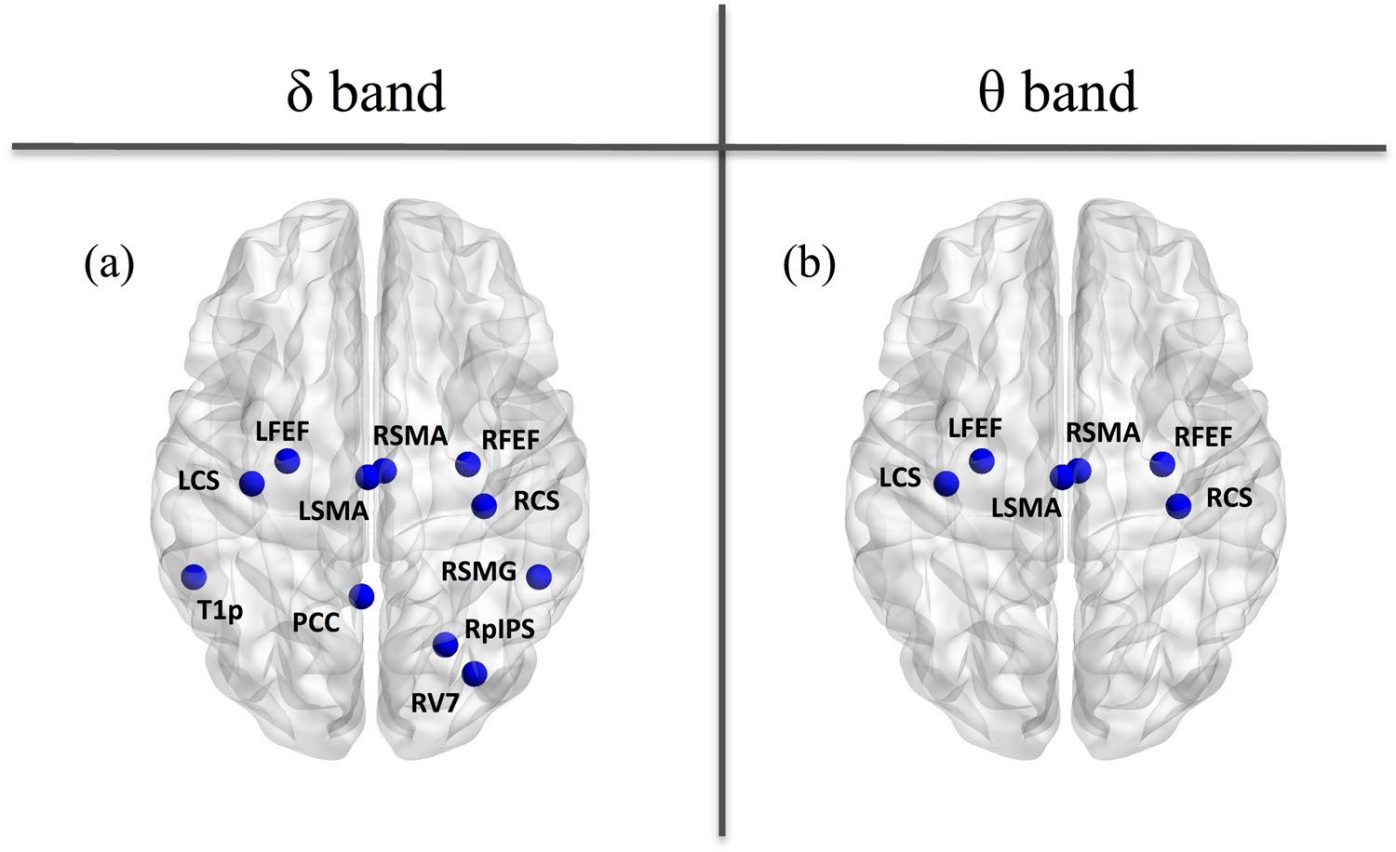
Significant age-related power decreases from young to elder subjects in the δ band (left) and the θ band (right) for the static analyses. The nodes are displayed onto the glass MNI brain.

As control analysis, we also considered static power changes in slow wave activity and only observed significant power decreases with aging in some nodes for the δ and the θ bands (Fig. 3). More precisely, we noted a power reduction for 3 nodes of the DAN, 1 node of the VAN, 1 node of the DMN, 1 node of the VISN, 4 nodes of the SMN and 1 node of the LAN in the δ band (Fig. 3a); and for 2 nodes of the DAN and 4 nodes of the SMN in the θ band (Fig. 3b).

The absence of age-related rsFC changes found in this study suggests that the electrophysiological connectome is actually maintained with age, contrary to what is claimed in the fMRI literature. However, one might argue that the FDR correction used in this study might be too conservative. Still, the massive number of comparisons involved (mainly due to the spatial degrees of freedom associated with the 40 nodes considered) cannot be left unhandled. Fortunately, the number of those spatial degrees of freedom can be estimated quite accurately, as it solely depends on the source reconstruction model, and this aspect can thus be taken into account in a precise manner^47^. So we also used an alternative approach whereby the family-wise error rate (FWER) associated with the spatial comparisons is corrected (in the spirit of random field theory for positron emission tomography and fMRI^48^ and analogs for MEG^49^) while the other factors remain uncontrolled, leading to a somewhat more liberal approach. In this case, only a few age-related FC changes could be observed on top of power changes, so that the conclusion that the electrophysiological connectome is globally maintained with age stands. (See Supplemental statistical analysis, Table S2 and Figure S1 for details).

## Discussion

The main purpose of this work was to investigate using MEG connectivity approaches whether aging induces modulations of the human functional brain architecture. To that aim, we first rigorously selected a population of elder participants, in order to avoid any bias related to the use of psychotropic drugs, the presence of recognized sleep impairment, and to psychiatric or cognitive confounding factors. Also, MEG allowed bypassing the neurovascular coupling issue typically encountered when using fMRI. Finally, we used the power envelope correlation approach to index rsFC together with a connectome approach allowing the investigation of within- and between-RSNs interactions to make a link with the available fMRI literature. Indeed, previous studies suggest that this MEG connectivity index allows uncovering similar RSNs as fMRI^37,39^. Results showed that no functional connection (either within or between networks) appeared modulated by aging. These results therefore suggest that the electrophysiological connectome is maintained in healthy elders. This observation contrasts with the available fMRI literature demonstrating substantial changes in functional connectivity with healthy aging.

Methodologically, we controlled the FDR to take into account the problem of multiple comparisons inherent to such neuroimaging data analyses. Nonetheless, one might argue that FDR might be too conservative and, consequently, that our negative results are due to too strict statistics. However, a more liberal approach based on spatial FWER control highlighted subtle rsFC changes at best; leaving our conclusion unchanged. Another possible issue might be a lack of statistical power associated with the relatively low number of subjects included. However, previous fMRI studies that disclosed significant and distributed age-related changes in rsFC included a similar (or even lower) number of subjects as the one used in this study, i.e., 25 subjects in each group (see, e.g.^50–56^). Still, increasing this sample size might help detecting possible age-related effects, but it is noteworthy that the drastic screening of elders used in this study renders this task rather difficult.

We used the power envelope correlation approach as rsFC index because it provides an electrophysiological equivalent to the fMRI RSNs^37^, at least for young healthy adults. So, why do the results of this MEG study therefore contrast with previous fMRI studies that disclosed significant changes in static within- and between-RSNs interactions between young adults and elder subjects? The discrepancy identified here between our MEG data and the fMRI literature with regard to aging is possibly rooted in two different factors. First, fMRI indirectly records neuronal activity via the neurovascular coupling, which is presumably altered by age (for a review, see, e.g.^34^). Thus, this study might suggest that the age-related modifications in rsFC previously reported using fMRI could simply represent an epiphenomenon (i.e., age-related changes in neurovascular coupling) rather than being directly relevant to brain networks, as already suggested in the fMRI literature (see, e.g^50^). Second, as already stressed above, the study of the effects of aging on the functional brain organization is typically associated with several possible confounding factors (e.g., sleep problems, psychotropic drugs, psychiatric or cognitive comorbidities) when it turns to the comparison of elders with young healthy adults. This is why, in this study, elder subjects were thoroughly screened for those confounding factors to leave age as much as possible as the main discriminant factor between the two populations. One possible drawback of such approach is that the included elder subjects might actually not be considered as representative of “typical” elders. As a matter of fact, based on the selection criteria adopted, this study tested subjects representative of elders with healthy or successful aging, as far as those concepts may be defined and delineated (for a review, see, e.g.^57^). This hypothesis is reinforced by the observed age-related decrease in the power of slow brain activity, i.e., the δ and θ bands. Indeed, previous studies have shown that power increases in those frequency bands with aging are typically associated with cognitive decline, while power decreases are associated with healthy or successful aging^43–46^. Still, in a fMRI study where elder subjects were thoroughly screened for confounding psychiatric and cognitive factors (including a comprehensive neuropsychological evaluation), researchers identified significant age-related changes in within and between networks rsFC^50^. Therefore, using similar populations, fMRI and MEG resting-state investigations might lead to different findings regarding the effects of healthy aging on functional brain integration. Our MEG data together with other neuroimaging studies supporting the “brain maintenance” theory for healthy or successful aging^6^ suggest that preserved structural and functional brain architecture may actually contributes to preserved cognitive functioning in healthy elders. Based on the above considerations, this study also highlights the critical need to compare fMRI and MEG rsFC changes with age in the same population of subjects and in elder subjects with different behavioral and cognitive profiles to provide further evidence supporting this hypothesis. Existent multimodal neuroimaging data repository such as the Cambridge Centre for Aging and Neuroscience (Cam-CAN) data repository^58^, might represent a unique opportunity to perform such comparison.

Finally, the results of this study pave the way for the use of MEG and power envelope correlation for the proper investigation of pathological aging pathophysiology and for the comparison with the fMRI literature on that topic. Indeed, the fact that we failed to find substantial MEG rsFC changes in healthy elders compared to young adult subjects together with the finding that patients with amnestic mild cognitive impairment show significantly altered static within-DMN rsFC (using power envelope correlation) compared with age-matched healthy controls^59^, suggest that the approach proposed in this paper would be sensitive to the disruptions in functional brain integration associated with pathological aging. The rationale for using such functional connectivity approach is also in line with the more advanced theories about Alzheimer’s disease (AD) pathophysiology, positing that amyloid-β and tau protein deposition progressively induces specific brain networks dysfunction due to (i) deficits in synaptic plasticity leading (together with other pathological processes) to neuronal hyperactivity, and (ii) the propagation of those proteins deposition at downstream projection structures (possibly in a prion-like manner) contributing to their sequential appearance in regions constituting those specific networks (for reviews, see, e.g.^60^). Also, considering that the vascular changes associated with AD and preclinical AD, which may affect the neurovascular coupling^61,62^, using a neuroimaging method free of the neurovascular coupling issue will be of utmost interest to address those questions.

To sum up, we have shown here using MEG that healthy aging does not induce marked changes in the functional organization of the human brain at rest. This study - and its natural prolongations suggested above - represents a first step towards a firm basis for the application of MEG rsFC relying on  power envelope correlation and its comparison with fMRI data for the characterization of the brain networks dysfunctions associated with pathological aging.

## Materials and Methods

### Participants and  screening

Twenty-five young (12 females and 13 males; age: 23.6 ± 2.9 years (mean ± standard deviation); age range: 19–31 years) and twenty-five elderly (15 females and 10 males; 68.8 ± 2.4 years; age range: 65–74 years) healthy adult subjects were included in this study. Of notice, resting-state MEG data from fifteen of the twenty-five elders were already used in previous studies from our group^63,64^. All participants were right-handed according to the Edinburgh handedness questionnaire (laterality scores young: 76.4 ± 15.2; laterality scores old: 89.2 ± 13.4), had no prior history of neurological or psychiatric disorder, and did not report of any subjective sleep or cognitive (e.g., memory impairment) problem. Elders had an active personal and social life and were thoroughly screened for psychotropic drug intake as well as for sleep habits, depression, anxiety and objective signs of pathological cognitive decline. The fifteen elders that contributed to^63^ were screened for (1) depression with the Short version of the Beck Depression Inventory^65^ (French adaptation by^66^), (2) anxiety with the State-Trait Anxiety Inventory (STAI, French version of ^67^), and (3) dementia with the Mattis Dementia Rating Scale^68^. The additional ten elders were screened for (1) depression with the Geriatric Depression Scale^69^, (2) anxiety with the State-Trait Anxiety Inventory (French version of ^67^), and (3) dementia using the Montreal Cognitive Assessment^70^. Those ten subjects also underwent a comprehensive neuropsychological evaluation in which (i) episodic memory was assessed using the Grober and Buschke’s procedure^71^, (ii) short-term memory, using the Forward Digit span (WAIS-III^72^) and the Block tapping test^73^, (iii) working memory, using the Backward Digit span (WAIS-III^72^), (iv) visuospatial processing, using the Rey-Osterrieth complex figure^74^, (v) language functioning, using the verbal fluency test^75^, and (vi) executive functions, with the Trail Making Test (parts A and B^76^), the Tower of London^77^, the Wisconsin Card Sorting Test^78^ and the color-word Stroop test^79^. All those ten elder subjects scored within the normal range for their age and level of education in all neuropsychological tests (see Supplementary Table S3). Table 1 summarizes the demographic information of the elder participants as well as the resulting scores. For each test/scale used, the inclusion scores shown in the table indicate that all elders were free from depression, anxiety and pathological cognitive decline. The education level as calculated on the basis of the International Standard Classification of Education was similar in young (4.8 ± 1.9; range: 2–7) and elder (5.1 ± 2.1; range: 2–8) participants (Student’s t-test: t_48_ = −0.49, p = 0.62, two-sided unpaired). Finally, sleep habits on the previous month as measured with the Pittsburgh Sleep Quality Index (PSQI^80^) were similar between young (3.8 ± 2.4; range: 1–12) and elder (3.2 ± 2.2; range: 0–10) subjects (Student’s t-test: t_48_ = 1.04, p = 0.3, two-sided unpaired).

Each participant contributed to the study after written informed consent. The CUB-Hôpital Erasme Ethics Committee approved this study prior to participants’ inclusion. All experiments were performed in accordance with relevant guidelines and regulations.

### MEG data acquisition and structural MRI

Neuromagnetic brain activity was recorded at rest (5 minutes, eyes open, fixation cross, sampling frequency: 1 kHz, online band-pass filter: 0.1–330 Hz) with a 306-channels whole-scalp MEG system installed in a light-weight magnetically shielded room (Vectorview & Maxshield; Elekta Oy; Helsinki, Finland; see^81^ for a description of its characteristics). Four head tracking coils continuously monitored subjects’ head position inside the MEG helmet. Coils’ location and at least 200 head-surface points were determined with respect to anatomical fiducials with an electromagnetic tracker (Fastrak, Polhemus, Colchester, Vermont, USA). Participants’ high-resolution 3D-T1 cerebral magnetic resonance images (MRIs) were acquired on a 1.5 T MRI scanner (Intera, Philips, The Netherlands).

### Data preprocessing

Firstly, the signal space separation method^82^ was applied off-line to the continuous MEG data to reduce external magnetic interferences and correct for head movements. Secondly, ocular, cardiac and system artifacts were eliminated using an independent component analysis^83^ (FastICA algorithm with dimension reduction to 30 components; hyperbolic tangent nonlinearity function) of the filtered data (off-line band-pass filter: 0.1–45 Hz). The components corresponding to artifacts were identified by visual inspection.

To proceed towards source reconstruction, the MEG forward model was also computed on the basis of participants’ MRI, which was anatomically segmented beforehand using the FreeSurfer software (Martinos Center for Biomedical Imaging, Massachussetts, USA). MEG and MRI coordinate systems were co-registered using three anatomical fiducial points for initial estimation and the head-surface points to manually refine the surface co-registration. Then, a cortically-constrained grid of dipole locations (mean inter-sources distance: 5 millimeters) was built in the Montreal Neurological Institute (MNI) template using the MNE suite (Martinos Centre for Biomedical Imaging, Massachussetts, USA) and non-linearly deformed onto each participant’s MRI with Statistical Parametric Mapping (SPM8, Wellcome Trust Centre for Neuroimaging, London, UK). The forward model associated with this source space was computed using a one-layer Boundary Element Method as implemented in the MNE suite.

### MEG source reconstruction

The following in-house pipeline was used for source reconstruction and envelope connectivity analysis (for more details, see^47^). Cleaned MEG data were filtered in the delta (δ band: 1–3 Hz), theta (θ band: 4–7 Hz), alpha (α band: 8–13 Hz) and beta (β band: 13–25 Hz) frequency bands. Band-specific Minimum Norm Estimation (MNE^84^) based on planar gradiometers only was then applied to reconstruct the sources of band-limited activity. Here, the noise covariance matrix was estimated from 5 minutes of empty-room data filtered in the relevant frequency range, and the regularization parameter was estimated using the consistency condition derived in Wens *et al*.^47^. The depth bias was corrected by a noise normalization scheme, i.e., dynamic statistical parametric mapping (dSPM)^84^. Three-dimensional dipole time series were projected on their direction of maximum variance, and the analytic source signals were finally extracted using the Hilbert transform.

###  Resting-state functional connectivity

Instead of working with seed-based connectivity maps, which often limit investigations to one or few networks, we focused here on a connectome approach. We selected forty cortical source locations taken from six well-known RSNs; the MNI coordinates of which were taken from^41^ (see Supplementary Table S1 in^41^). Seven nodes were located in the DMN, six in the DAN, five in the VAN, ten in the VISN, six in the SMN, and six in the LAN (see Fig. 1). Forty-by-forty matrices of rsFC were built by computing the slow envelope correlation (see, e.g.^39^) between (i) the associated forty source signals (each viewed as a seed signal) and (ii) the same signals (viewed as targets) corrected beforehand for spatial leakage from the seed. Spatial leakage indeed leads to strong spurious connectivity that dominates over physiological couplings and was corrected here using the geometric correction scheme^47^. Because spatial leakage correction induces slight asymmetries between seed and target (notwithstanding genuinely symmetrical approaches such as^85^), the resulting rsFC matrices were symmetrized afterwards. We also computed forty-by-one vectors containing the power estimate (i.e., source signals’ variance) at each node to control for possible power-induced effects in rsFC changes.

The aforementioned pipeline was applied either on the entire timespan of the recording for the static rsFC analysis, or on moving windows (length: 10 seconds, step: 2 seconds; parameters based on^41^) for the dynamic rsFC analysis. The individual output data were gathered into a forty-by-forty-by-*W* rsFC array and a forty-by-*W* power matrix; *W* indicating the number of time windows (static case: *W* = 1; dynamic case: *W*≈150). The temporal fluctuations of dynamic rsFC patterns are generally quite complex^86^ and their analysis typically relies on pattern classification methods such as maximum correlation windows^40,41^ or clustering of rsFC states^42,87,88^. Here, we rather focused on the basic question of whether the temporal variability of rsFC time series was affected by physiological aging. Therefore, we considered the SD and the CS (i.e., mean-over-SD) of the dynamic rsFC data, both computed in each individual across windows.

This presented pipeline has been entirely applied to the α and β bands whereas only static power was computed for the δ and θ bands.

### Statistical assessments

To assess the effect of age on each type of output data (static, SD and CS of rsFC/power), we computed group-averaged differences between the two populations and derived *p*-values using standard non-parametric unpaired, two-tailed permutation tests (10^6^ random permutations of the age condition, see, e.g.^89^). The significance level at *p* < 0.05 was corrected to take into account the massive number of comparisons involved, i.e., the spatial factor based on the number of cortical nodes investigated (40 × 39/2 = 780 connections for rsFC and 40 nodes for power) and the non-spatial factors comprising the frequency bands (delta, theta, alpha and beta), static and dynamic indices (SD and CS), and rsFC and power. The correction was based on the Benjamini-Hochberg algorithm^90^ in order to control the FDR. Another approach controlling the FWER for the spatial degrees of freedom only is described in the Supplementary Materials (see Supplemental statistical analysis).

### Data availability

The datasets analyzed during the current study are available from the corresponding author upon reasonable request.

## Electronic supplementary material

Supplementary Materials
