# Supplementary material for: The electrophysiological connectome is maintained in healthy elders: a power envelope correlation MEG study
Source: Sci Rep. 2017 Oct 25;7:13984. doi: 10.1038/s41598-017-13829-8 (PMC5656690; doi:10.1038/s41598-017-13829-8)
Supplement: Supplementary file 1 — Supplementary Materials [file 41598_2017_13829_MOESM1_ESM.pdf]

**The electrophysiological connectome is maintained in healthy elders : a power envelope correlation MEG study**

***Supplementary Materials***

Coquelet N[1*], Mary A[2,3], Peigneux P[2], Goldman S[1,4], Wens V[1,4°], De Tiège X[1,4°]

[1] Laboratoire de Cartographie fonctionnelle du Cerveau (LCFC), UNI – ULB Neuroscience Institute, Université libre de Bruxelles (ULB), Brussels, Belgium.

[2] Neuropsychology and Functional Imaging Research Group (UR2NF), Centre for Research in Cognition and Neurosciences (CRCN), UNI – ULB Neuroscience Institute, Université libre de Bruxelles (ULB), Brussels, Belgium.

[3] Normandie Univ, UNICAEN, PSL Research University, EPHE, INSERM, U1077, CHU de Caen, Neuropsychologie et Imagerie de la Mémoire Humaine, Caen, France.

[4] Department of functional Neuroimaging, CUB Hôpital Erasme, Université libre de Bruxelles, Brussels, Belgium.

° These authors equally contributed to the paper

**Corresponding author** Nicolas Coquelet, Laboratoire de Cartographie fonctionnelle du Cerveau, UNI – ULB Neuroscience Institute, Université libre de Bruxelles (ULB), 808 Lennik Street, 1070 Brussels, Belgium. Tel : +32 555 42 98, Fax : +32 555 66 31, E-mail : ncoquele@ulb.ac.be

## Supplemental statistical analysis

As the false discovery rate (FDR) procedure used to correct for multiple comparisons may lead to conservative detection of significance, we repeated our analyses using an alternative method controlling the spatial family wise error rate (FWER) accurately but does not take the other comparison factors into account, leading to a somewhat liberal detection of significance.

We adapted the approach of [47] whereby the effective number $N_{\mathrm{eff}}$ of independent nodes (for the power analyses) or connections (for the rsFC analyses) is assessed directly, so that corrected $p$-values are derived using Bonferroni correction (i.e., $p^{\mathrm{corr}} = N_{\mathrm{eff}} \times p^{\mathrm{uncorr}}$). Briefly, two sources taken among the 40 nodes of interest may share partial information because of spatial leakage, which effectively decreases the number of comparisons[47]. Alongside similar ideas underlying random field theory in SPM[48] or analogs for electromagnetic responses[49], we estimated here the actual number $\rho$ of spatial degrees of freedom emerging from the 40 cortical nodes of interest reconstructed via MNE. Specifically, $\rho$ was derived as the rank of the forward model (see [47] for details) restricted beforehand to the 40 nodes. For the present data, $\rho = 21$ so the Bonferroni correction factor was $N_{\mathrm{eff}} = \frac{\rho(\rho-1)}{2} = 210$ for the symmetrical rsFC matrices and $N_{\mathrm{eff}} = \rho = 21$ for the power vectors. A difference in a given rsFC or power entry was thus deemed significant whenever the corresponding $p$-value satisfied $p^{\mathrm{corr}} < 0.05$. The non-spatial factors were left uncorrected since it is difficult to assess a priori their interdependence. Indeed, a further naïve Bonferroni correction for the non-spatial factors would lead us again to a too conservative setup. The price to pay is that this approach may be too lenient, and the truth must lie in between the FDR and this spatial FWER analyses.

Fortunately results converged, at least qualitatively. Indeed only a few band-specific

rsFC entries (less than 1%) disclosed significant age-related changes (see Figure S1 and Table S2). In the static estimates (Figure S1a,b), no age-related differences were found in α-band rsFC while one increased cross DMN-VAN connection was identified in the β band. For the dynamic SD across short time windows (Figure S1c,d), no significant age-related change was observed for both α and β bands while for the CS (Figure S1e,f), age-related changes were found in the β band only: rsFC decreases for one connection within the DMN and one cross-RSNs connection between the VISN and SMN networks, and rsFC increases for two connections within the VISN and one cross-RSNs connection between VISN and DMN.

Regarding the age-related power modulations in the α and β bands, we also observed significant entries (Figure S1). For the static evaluation (Figure S1a,b), significant power decreases is found from young adults to elders in one node of the DAN in the α band, one node of the VAN and two nodes of the SMN; for the β band, power increases in a different node of the DMN and a node of the LAN. For the dynamic SD (Figure S1c,d), significant age-related power decreases were observed for two SMN nodes in the α band and for two VISN nodes in the β band, and power increases for one SMN node and one LAN node in the β band. For the CS (Figure S1e,f), a power age-related changes decrease in one node of VISN was found in the β band. It is also noteworthy that none of the age-related differences between power and rsFC estimates overlapped, indicating that possible rsFC changes were not induced by power changes.

Finally, regarding the age-related changes in the power of slow wave brain activity, we noted a power reduction for 3 nodes of the DAN, 1 node of the DMN, 2 nodes of the SMN and 1 node of the LAN in the δ band; and for 2 nodes of the DAN and 1 node of the SMN in the θ band.

## Supplemental Figures and Tables

| Static α | p-value |
|---|---|
| Right frontal eye field (DAN) | 0.0018 |
| Right pre-central sulcus (VAN) | 0.0011 |
| Right supplementary motor area (SMN) | 0.0001 |
| Left supplementary motor area (SMN) | 0.00003 |
| Right central sulcus (SMN) | 0.0055 |
| Static β | |
| Left second somatosensory (SMN) | 0.0053 |
| Left inferior temporal gyrus (DMN) | 0.0013 |
| Anterior superior temporal gyrus (LAN) | 0.0018 |
| Right area V4 (VISN) | 0.0058 |
| Dynamic α : standard deviation | |
| Right supplementary motor area (SMN) | 0.0003 |
| Left supplementary motor area (SMN) | 0.0004 |
| Dynamic β : standard deviation | |
| Pars triangularis Opercularis of the inferior frontal gyrus (LAN) | 0.0059 |
| Upper part of the pars Opercularis of the inferior frontal gyrus (LAN) | 0.0055 |
| Anterior superior temporal gyrus (LAN) | 0.0011 |
| Left inferior temporal gyrus (DMN) | 0.0025 |
| Left second somatosensory (SMN) | 0.0014 |
| Left area V3 (VISN) | 0.0019 |
| Right area V2 dorsal (VISN) | 0.0007 |
| Left area V2 dorsal (VISN) | 0.0044 |
| Dynamic α : coefficient of stability | |
| Right area V3 (VISN) | 0.0063 |
| Right area V2 dorsal (VISN) | 0.0024 |
| Left area V2 dorsal (VISN) | 0.0026 |
| Dynamic β : coefficient of stability | |
| Upper part of the pars Opercularis of the inferior frontal gyrus (LAN) | 0.0039 |
| Right ventral frontal cortex (VAN) | 0.0054 |
| Right area V2 dorsal (VISN) | 0.0015 |

**Table S1 : p-values for statistically significant nodes according to the FDR correction (corresponding to uncorrected $p<0.0066$) both static and dynamic analysis in the α and β bands. MNI coordinates can be found in [41].**

| | Static α | p-value |
|---|---|---|
| power | Right frontal eye field (DAN) | 0.0386 |
| | Right pre-central sulcus (VAN) | 0.0237 |
| | Right supplementary motor area (SMN) | 0.0007 |
| | Left supplementary motor area (SMN) | 0.0023 |
| | Static β | |
| power | Left inferior temporal gyrus (DMN) | 0.0271 |
| | Anterior superior temporal gyrus (LAN) | 0.0383 |
| rsFC | Precuneus (DMN) – Right middle frontal gyrus (VAN) | 0.0336 |
| | Dynamic α : standard deviation | |
| power | Right supplementary motor area (SMN) | 0.0059 |
| | Left supplementary motor area (SMN) | 0.0083 |
| | Dynamic β : standard deviation | |
| power | Left second somatosensory (SMN) | 0.0296 |
| | Anterior superior temporal gyrus (LAN) | 0.0230 |
| | Left area V3 (VISN) | 0.0396 |
| | Right area V2 dorsal (VISN) | 0.015 |
| | Dynamic α : coefficient of stability | |
| power | Right area V2 dorsal (VISN) | 0.0496 |
| | Dynamic β : coefficient of stability | |
| power | Right area V2 dorsal (VISN) | 0.0314 |
| rsFC | Left area V2 dorsal (VISN) – left area V1 (VISN) | 0.0363 |
| | Left area V7 (VISN) – left area V1 (VISN) | 0.0382 |
| | Left inferior temporal gyrus (DMN) – Left angular gyrus (DMN) | 0.0168 |
| | Precuneus (DMN) – Left area V1 (VISN) | 0.0397 |
| | Left second somatosensory (SMN) – Left area V7 (VISN) | 0.0443 |

**Table S2 : p-values corrected for spatial comparisons using FWER for both static and dynamic analysis in the α and β bands. MNI coordinates can be found in [41].**

| Neuropsychological tasks | Means (standard deviations) |
| --- | --- |
| Episodic memory (Grober and Buschke task) | |
| Last immediate free recall | 13.4 (2.22) |
| Last immediate cued recall | 16 (0) |
| Delayed free recall | 13.2 (1.75) |
| Delayed cued recall | 15.9 (0.32) |
| Short-term memory | |
| Forward Digit span | 5.7 (1.06) |
| Backward Digit span | 4.2 (1.14) |
| Block Tapping test | 5.1 (0.74) |
| Tower of London | |
| Total number of exceeding movements | 21.4 (13.57) |
| Total latencies | 86.8 (61.8) |
| Verbal fluency | |
| Phonemic (letter P) | 25.6 (5.36) |
| Semantic (animals) | 38.9 (11.03) |
| Trail Making test (TMT) | |
| TMT A (time) | 28.33 (3.12) |
| TMT A (errors) | 0 (0) |
| TMT B (time) | 57.24 (12.85) |
| TMT B (errors) | 0.2 (0.63) |
| Wisconsin Card Sorting test | |
| Total errors | 1 (1.41) |
| Stroop test | |
| Time interference index[1] | 0.23 (0.06) |
| Copy of the Rey Complex figure | |
| Time | 203.1 (169.23) |
| Score | 34.4 (1.17) |

**Table S3: Neuropsychological evaluation results for the elders' group (Group 2).**

[1] The time interference index in the Stroop is the time necessary to perform the interference condition subtracted by the time to perform the color naming condition, divided by the sum of both variables.

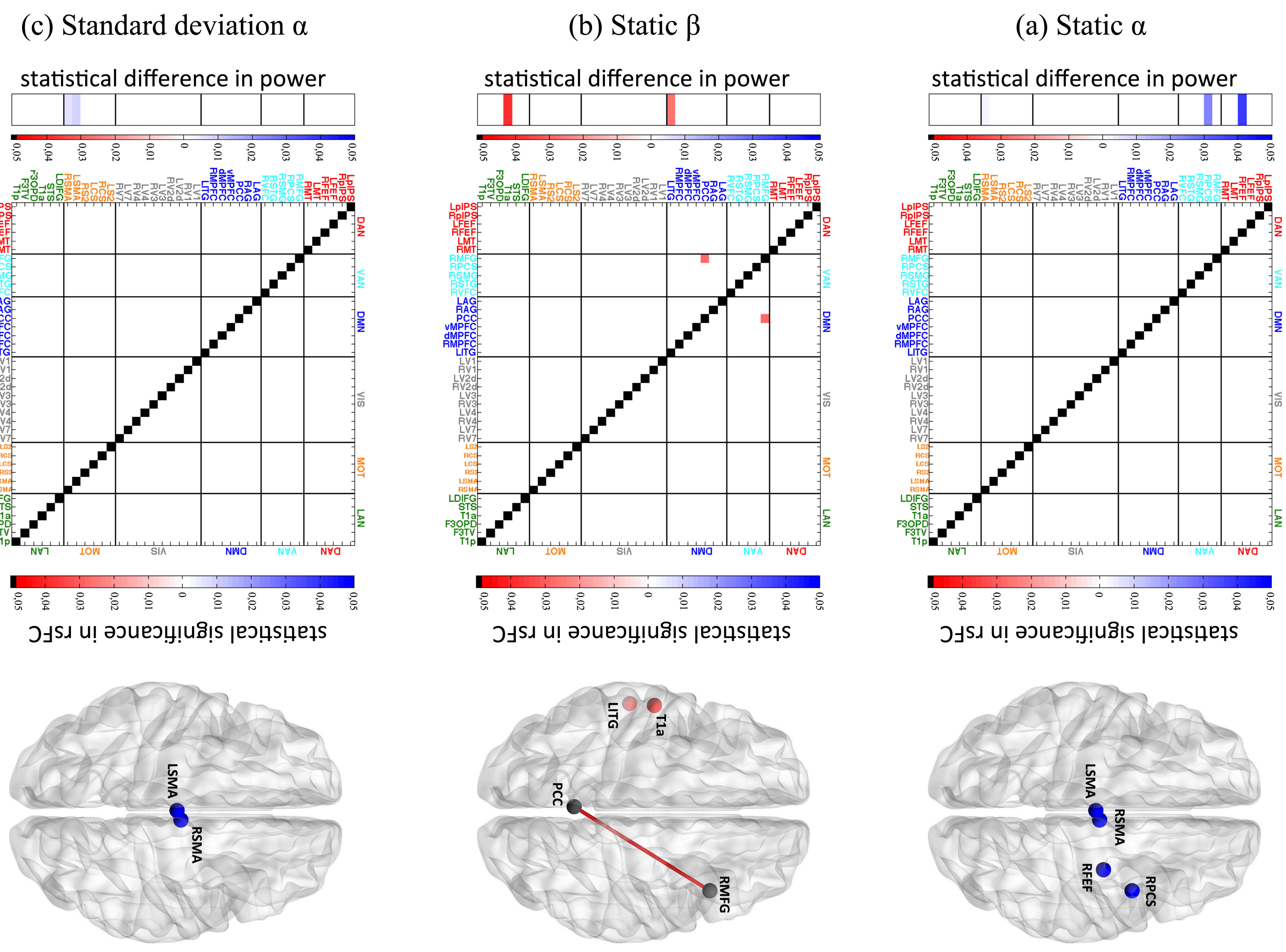
(a) Static α
statistical difference in power
statistical significance in rsFC
LSMA
RSMA
RFEF
RPCS
(b) Static β
statistical difference in power
statistical significance in rsFC
LITG
T1a
PCC
RMFG
(c) Standard deviation α
statistical difference in power
statistical significance in rsFC
LSMA
RSMA
DAN
VAN
DMN
VIS
MOT
LAN

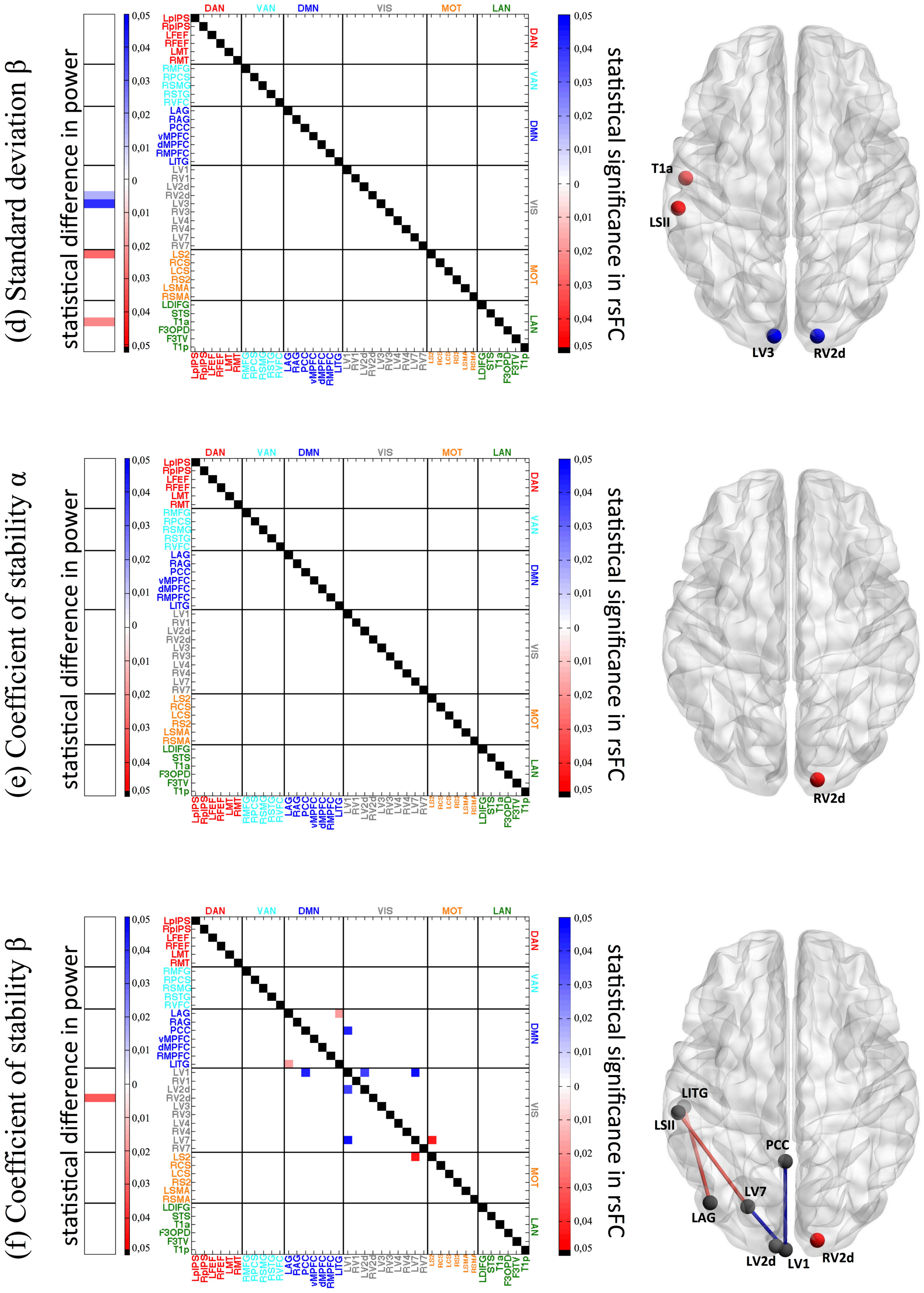


**Figure S1 : Statistical power and rsFC results for spatial comparisons corrected using the FWER technique. The left part discloses the corrected p-values of significant power (column) and rsFC (matrix) changes. The right part indicates their localization on the glass MNI brain. Color code for statistical analysis: blue corresponds to statistically higher values for young adults compared to healthy elders, while red ones corresponds to the contrary (healthy elders>young adults). Gray nodes are involved in rsFC modulations without power changes. MNI coordinates and labels can be found in [41].**
